# A Comprehensive Model of the Spatio-Temporal Stem Cell and Tissue Organisation in the Intestinal Crypt

**DOI:** 10.1371/journal.pcbi.1001045

**Published:** 2011-01-06

**Authors:** Peter Buske, Jörg Galle, Nick Barker, Gabriela Aust, Hans Clevers, Markus Loeffler

**Affiliations:** 1 Interdisciplinary Centre for Bioinformatics, University Leipzig, Leipzig, Germany; 2 Hubrecht Institute, University Medical Center, Utrecht, The Netherlands; 3 Center of Surgery, Research Laboratories, University Leipzig, Leipzig, Germany; 4 Institute for Medical Informatics, Statistics and Epidemiology, University Leipzig, Leipzig, Germany; Massachusetts Institute of Technology, United States of America

## Abstract

We introduce a novel dynamic model of stem cell and tissue organisation in murine intestinal crypts. Integrating the molecular, cellular and tissue level of description, this model links a broad spectrum of experimental observations encompassing spatially confined cell proliferation, directed cell migration, multiple cell lineage decisions and clonal competition.

Using computational simulations we demonstrate that the model is capable of quantitatively describing and predicting the dynamic behaviour of the intestinal tissue during steady state as well as after cell damage and following selective gain or loss of gene function manipulations affecting Wnt- and Notch-signalling. Our simulation results suggest that reversibility and flexibility of cellular decisions are key elements of robust tissue organisation of the intestine. We predict that the tissue should be able to fully recover after complete elimination of cellular subpopulations including subpopulations deemed to be functional stem cells. This challenges current views of tissue stem cell organisation.

## Author Summary

In the murine small intestine there are more than a million organized groups of proliferating cells, the crypts, each of which contains about 250–300 cells. About 60% of these cells are in rapid cycle. The functional stem cells of this tissue have been demonstrated to reside at defined positions at the lower third of the crypt and to give rise to four different cell types. Considering this simple structure the murine intestine is an ideal system to study general aspects of tissue organization. Here, we introduce a comprehensive and predictive computer model of the spatio-temporal organization of the murine intestine which describes how cell production and cell fate decisions could be organized in steady state as well as under perturbations. The model is based on single cells acting as individual agents, updating their status within a certain set of options governed by some active rules and on signals received from the environment. This kind of self-organization enables effective tissue regeneration without assuming an explicit stem cell population that maintains itself by asymmetric division. Thus, the model offers a novel systems biological view on crypt stem cell and tissue organisation.

## Introduction

**Figure 1:**
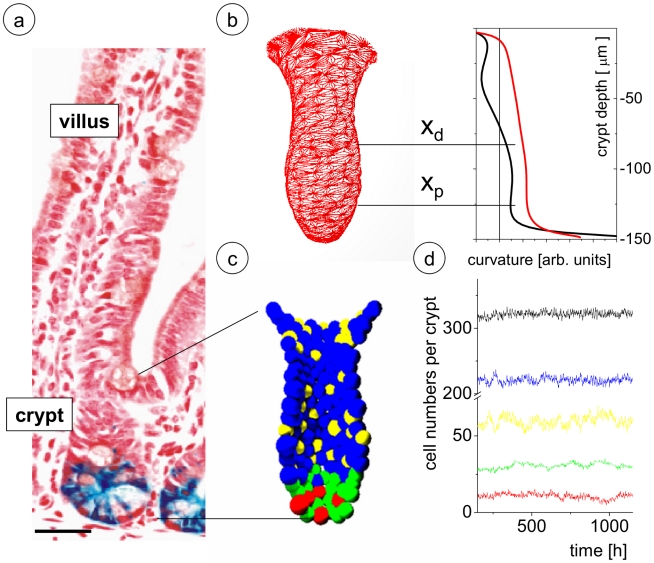
Model of the murine small intestinal crypt. a) Histological section. Expression of the functional stem cell marker Lgr5-LacZ (blue) is mainly restricted to a few cells at the crypt bottom [6] Bar: 50µm. b) Example of a model network representing the BM of the crypt. The Gaussian (black) and Mean (red) curvature [59] are highest at the crypt bottom. Wnt- activity is assumed to correlate with curvature and to adopt threshold values at position x_p_ and x_d_ (see text). c) Snapshot of a crypt simulation. Undifferentiated cells (red) and Paneth cells (green) are found intermingled at the crypt bottom, progenitors of enterocytes (blue) and Goblet cells (yellow) move upwards along the crypt axis. d) Steady state cell numbers over time. Colour code as in c). Black line denotes the total number of cells.

The epithelium of the small intestine is the most rapidly regenerating tissue of adult mammals. Cell production starts near the crypt base, producing numerous progeny which move up the crypt-villus axis. Cells moving up the crypt continue proliferating while in parallel becoming committed either to an absorptive or a secretory fate. Cells stop proliferating and differentiate while approaching the crypt-villus junction. Upon reaching the villus tip a few days later cells are shed into the lumen of the intestine. As an exception, cells that become committed to the secretory Paneth lineage move down the crypt-villus axis whilst differentiating until they occupy their final position at the very bottom of the crypt. These cells have a life time of up to 8 weeks [1]. A histological section through this system is shown in Fig. 1a.

Tissue and stem cell organisation of the adult small intestine has been studied extensively [2], [3], [4]. There are indications that functional intestinal stem cells are localized in specific niche positions characterized by specific markers [5]. Lgr5 and Bmi1 expression were both demonstrated to be features of cells capable of self-renewal and of generating differentiated progeny [6], [7] hence fulfilling the functional stem cell criterion [8]. Additional markers have been suggested [9], [10]. The relationship between these cellular phenotypes and the stem cell functionality is presently not fully understood.

The nature of the microenvironment that harbours and possibly conditions the functional stem cells is also not fully elucidated. Activation of the Wnt- and Notch- pathway was demonstrated to be essential for stem cell maintenance as well as proliferation and differentiation [11], [12]. These experiments also showed that cell fate and cell lineage decisions can be rapidly and dramatically shifted by activating gain or loss of gene function in these signalling pathways. Such data implicate the idea of flexibly switching cell activity modes on and off by controlling the local signalling environment and hence challenge the classical view of tissue stem cell organisation based on a pedigree concept [8].

The classical ‘pedigree concept’ of hierarchical tissue organization regards ‘stemness’ as a cellular property essentially fixed intrinsically to specified cells called stem cells annotating them as a specific cell type. These cells are assumed to divide asymmetrically and to give rise to a new stem cell and to a non-stem progenitor cell. Subsequently, the progenitor cell undergoes transient amplifying divisions before it differentiates terminally. In this pedigree model lineage specification is linked to cell stages in the cellular differentiation hierarchy. It is one of our objectives here to demonstrate that the pedigree related assumptions on stem cell populations are not required in order to provide a comprehensive explanation of the tissue self organisation and that an alternative concept can be more powerful.

Our approach is based on concepts of self-organizing systems assigning a greater emphasis to the interaction between cells and their environment. Moreover, they enable reversible developments for individual cells, allowing the system to flexibly react to changing demands [13], [14], [15]. Applying these general concepts we designed a comprehensive and predictive 3D individual cell-based model of the organisation and cell turnover of murine intestinal crypts. The model includes representations of basic molecular regulation mechanisms of proliferation and lineage specification based on Wnt- and Notch-signalling and all major aspects of stem cell function. We use the model to describe steady state cell turnover and conversion dynamics of clonally labelled cells. Moreover, the lineage specification and cell differentiation within the crypt is described, as well as changes it undergoes subsequent to gain and loss of gene function and physical tissue damage.

## Results

### Model description

#### Cells

Cells are represented by elastic objects which can move, grow and divide, form contacts with other cells and the basal membrane (BM) and can communicate with one another. The extent by which these properties are expressed depends on the internal state vector 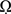 of each cell. An important feature covered by 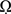 is the activity status, i.e. the transcription of target genes, of the Wnt- and Notch-pathway denoted by I_Wnt_ and I_Notch_, respectively.

#### Wnt-signalling

Wnt-signalling in intestinal crypts has been extensively studied [16]. The central player in the canonical Wnt-pathway is β-catenin. While β-catenin is targeted for proteosomal degradation in absence of a Wnt-signal, in presence of this signal it can accumulate in the cell. Coincident translocation of β-catenin into the nucleus results in binding to transcription factors of the TCF/LEF family and leads to activation of target genes. For a detailed description see e.g. [17]. Wnt-signalling in crypt cells was observed to be highest at the crypt bottom and to decrease continuously along the crypt-villus axis [18]. This Wnt-gradient is conserved during loss of cell sorting in the intestine, leading to the suggestions that its formation is essentially a non cell-autonomous process [19]. Using genetically engineered mice it has been shown that secretory cells are dispensable for differential Wnt-activity in the crypts [20], [21]. Recently, using *in vitro* culture of primary intestinal cells it has been demonstrated that stroma cells are not required for induction of this gradient and to generate functional tissue [22]. The same study demonstrates that the Wnt-activity gradient forms in crypts even if a constant high concentration of the Wnt agonist R-Spondin1 is provided to all cells. This led us to the hypothesis that the formation of the Wnt-activity gradient in the crypt is largely independent of the expression of Wnt-molecules. On the other hand positive surface curvature was demonstrated to induce growth activity in several epithelia [23], [24]. In crypts it is highest at the bottom cap (see Fig. 1b). We therefore assume in our model that Wnt-activity is determined by the local curvature of the basal membrane. Consequently, the internal state 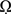 of the cell depends on the local crypt shape.

#### Notch-signalling

Notch-signalling is mediated via transmembrane proteins. Thus, it requires cell-cell contacts. It is activated in Notch-receptor expressing cells if their neighbour cells express Notch-ligands such as Jagged and Delta [25]. Upon receptor-ligand binding, Notch proteins are cleaved by γ-secretase and in turn the Notch intracellular domain translocates to the nucleus, where it activates transcription of target genes [26]. In the crypt undifferentiated cells and cells of the absorptive lineage and their progenitors produce Notch (van Es & Clevers, unpublished data). In contrast, cells of the secretory lineages and their progenitors are assumed to express Notch-ligands. A Notch-activated cell is prevented from changing into a secretory lineage as long as its neighbour cells express sufficient Notch-ligands. This phenomenon is known as lateral inhibition [27]. Accordingly, we assume that the internal state of a cell depends not only on the crypt shape but also on the number and types of cell-cell contacts formed.

#### Lineage specification and differentiation model

In our model, lineage specification into enterocytes (absorptive lineage) and Paneth- and Goblet cells (secretory lineages) is assumed to depend on Wnt- and Notch-signalling [16], [26]. For both pathways activity thresholds have been suggested (Wnt: [28], Notch: [29], [30]). Accordingly, and in order to enable a cell type classification we assume the following threshold-dependent cell fates:

Cells with high Wnt- and high Notch-signalling, i.e. with I_Wnt_ and I_Notch_ above certain thresholds TP_Wnt_ and TP_Notch_, respectively, are considered as undifferentiated ([16]. Notch-signalling was demonstrated to be required in order to avoid specification into a secretory lineage which seems otherwise to be a default mechanism [12], [31]. Thus, we assume the secretory phenotypes to be related to low Notch-activity. Accordingly, undifferentiated cells with high I_Wnt_ become primed for switching on secretory properties if I_Notch_ drops below TP_Notch_. This occurs if their neighbour cells do not express sufficient Notch-ligands. High Wnt-signalling is required for Paneth cell differentiation [21], [32]. Moreover, at low Wnt-activity Paneth cells decrease in number while Goblet cells appear to be unaffected [33]. Thus, undifferentiated cells with high I_Wnt_ become primed for switching on Paneth properties.

Enterocytes are characterised by a low Wnt-activity compared to undifferentiated cells [33]. Their fate vanishes at constitutive high Wnt [34]. As a consequence we assume that an undifferentiated cell with high I_Notch_ becomes primed for switching on enterocyte properties if I_Wnt_ drops below the threshold TP_Wnt_. This occurs if the cell reaches a position above x_p_ (Fig. 1b). In the line of arguments given for Paneth cell specification the enterocyte progenitor is switched to the Goblet property if in addition I_Notch_ falls below TP_Notch_. In accordance with the concept of self-organizing systems [13], [14], [15] we assume priming to be reversible in general. We allow for any transition between the undifferentiated and progenitor cells and between different progenitor cells.

A primed cell can subsequently develop towards and may eventually reach an irreversible (terminal) differentiation state. Enterocyte and Goblet progenitors migrate out of the crypts and turn into differentiated cells if they reach a position near the crypt-villus junction [17]. As they move in a Wnt-gradient, we assume that they become irreversibly differentiated and stop their proliferation if I_Wnt_ falls below a threshold TD_Wnt,_ which happens if the cells reach positions above x_d_ (Fig. 1b). Terminal differentiation of Paneth cells requires SOX9, a Wnt-target, and thus, high Wnt-activity [20], [35]. Thus, no change in the considered Wnt-activity status is required. We assume that a Paneth cell reaches a terminal differentiation state after finishing its current cell cycle.

#### Spatio-temporal self-organisation

In our model the activity state of a cell is determined by its local environment (see above). As a result each conformational change of a cell changes its activity state and those of its neighbours. Accordingly lineage specification and differentiation strongly depend on cell migration, cell adhesion and cell elasticity which affect the spatio-temporal organisation of the crypt. These biomechanical features are modelled using an established individual cell-based model of epithelia [36], [37]. In this model the biomechanical properties of the cells are described by a set of experimentally accessible parameters. Simulations of the model are performed as ‘experiments in silico’ to be directly comparable to experimental results.

We assume identical biomechanical properties in all differentiation states, except for the migration properties. These properties were suggested to be controlled by the expression of Eph2/Eph3 receptors and their ligand ephrin-B1 [19]. Here, we assume Paneth cells progenitors to actively move down the crypt, whereas all other primed cells move actively upwards towards the crypt-villus junction. Active motion was modelled assuming that a ‘migration force’ acts on each cell. Passive cell motion is driven by all deterministic forces acting on a cell [37]. These forces refer to cell adhesion, deformation and compression. Details regarding the underlying cell-cell interactions are described in the Materials and Methods section (A1).

Besides the cell properties, properties of the basal membrane (BM) of the crypt also impact the spatio-temporal organisation process. We explicitly model the BM by an artificial fibre network that interacts with each individual cell. The shape and the size of the network representing the basal membrane was chosen in order to fit experimental data on crypt geometry [8]. Details regarding the construction of the BM network and the cell interactions with it can be found in the Materials and Methods section (A2).

#### Proliferation and apoptosis

Whether a cell proliferates depends on its differentiation state. Undifferentiated cells in the crypt are capable of proliferation [6]. Migrating up the crypt and becoming enterocyte progenitors they exit the cell cycle only if they become terminal differentiated while reaching the upper part of the crypt [17]. In contrast crypt cells in general stop proliferation after getting primed for a secretory lineage [26]. In our model proliferation is regulated according to the same rules. In general cells finish their actual cell cycle before entering a terminal differentiated state.

If a cell starts cycling, the cycle will be finished independently of changes in the differentiation state. Cell growth is modelled assuming stochastic growth steps. This leads to Γ-distributed growth times, where a cell doubles its volume. In our simulations the average value was adapted to about 16 hours. If the cell reaches twice the initial (minimal) volume it divides into two daughter cells of equal volume. Cells that are sufficiently compressed by their neighbour cells stop volume growth due to contact inhibition of growth [36]. Cells are removed from the crypt system if they lose contact with the basal membrane. They are thought to undergo anoikis [38]. If cells cross the crypt-villus junction they are formally removed. This ensures zero pressure conditions at this boundary in the simulations. The villus is not modelled. Paneth cells are assumed to have a limited life-span of about 8 weeks [1]. Afterwards, they are removed from the crypt.

#### System dynamics

The system dynamics is described by equations of motion for each individual cell. Thereby, each update of the position and size of the cells potentially changes the internal state vector 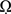 of each of them. Thus, 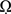 is updated subsequently. Details are found in the Materials and Methods section (A3). Due to the self-organisation properties of our model, changes of any model parameter have a complex effect on the systems behaviour. While the model behaviour was obtained to agree qualitatively with experimental findings in wide parameter ranges, quantitative agreement required detailed fitting. Insight into this procedure is given in the Materials and Methods section (A4).

### Simulation results

#### Modelling steady state cell production

**Figure 2:**
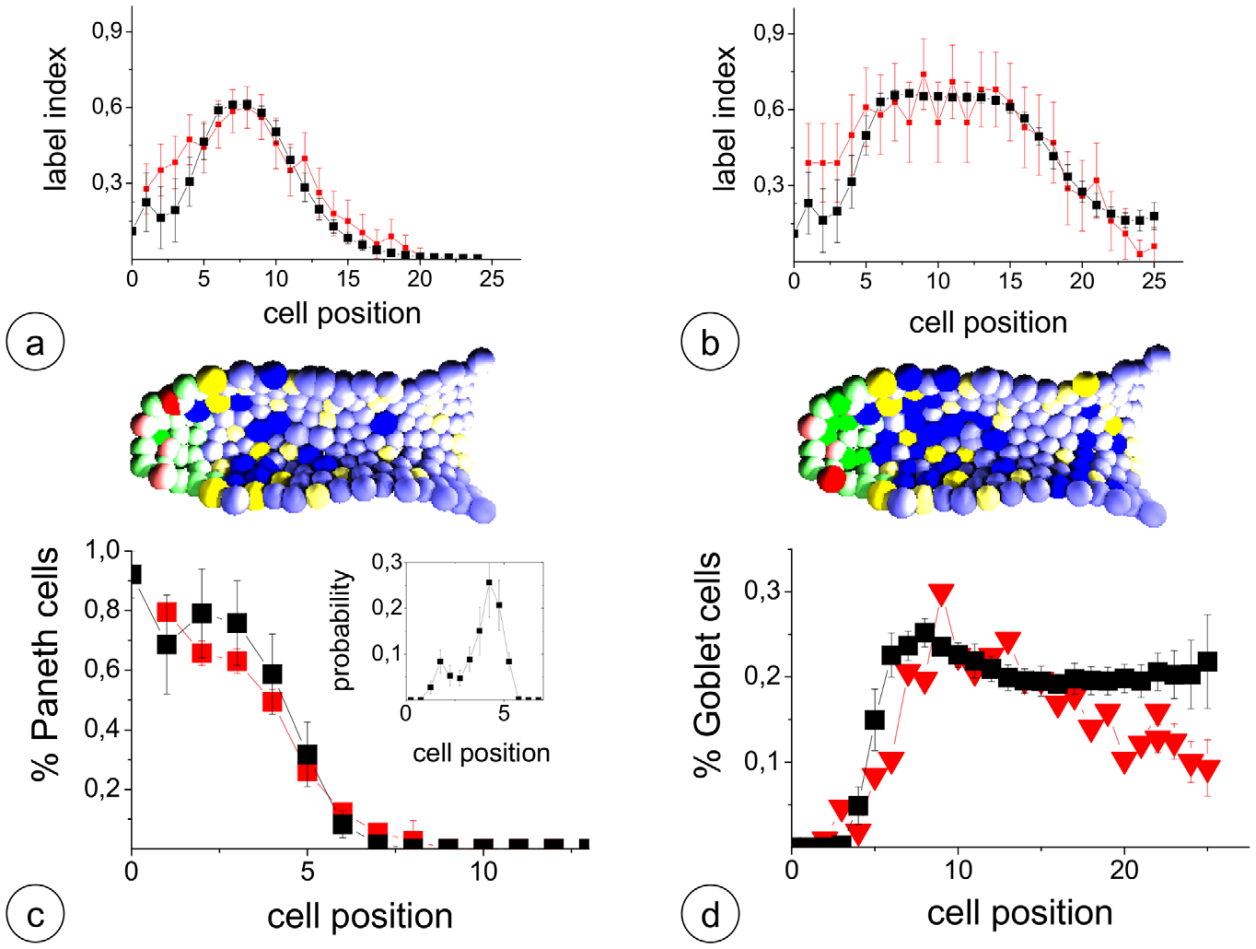
Steady state crypt. a,b) Positional BrdU label index obtained a) 2h and b) 24h after labelling. Experimental data: red, Simulation data: black. Bottom: Snapshots of simulated crypts. Colour code as in Fig. 1. Saturated colour indicates cells labelled for proliferation activity. c),d) Experimental and simulated distribution of c) Paneth, and d) Goblet cells in the crypt. Experimental data (red) were taken from [40] and [42], respectively. The insert in c) shows the simulated distribution of initial Paneth cell positions. A video showing an example of a steady state crypt simulation can be found in Video S1.

Our crypt model is capable of quantitatively reproducing steady state cell production (Fig. 1d). Thereby, the entire crypt has a turnover of about 2–3 days. The average spontaneous apoptosis rate within the crypt is below 5 per cent in agreement with results by Marshman et al. [39]. The parameter set used to obtain this dynamics is given in Table 1 (see Materials and Methods). It was obtained by fitting the model behaviour to data on the proliferation activity in murine intestinal crypts based on BrdU labelling experiments (details see Materials and Methods A5). The results of these experiments and their fits by our model are shown in Fig. 2a,b. A video of a crypt in steady state is shown in Video S1.

**Table 1:** Parameters of the model.

Symbol	Value	Parameter	Reference
**Parameter of the cell model**			
V_0_	4/3π (5µm)^3^	Minimal volume of an isolated cell	Estimated
τ	14 h	cell growth time	results in an effective cell cycle time ∼24h
E	1kPa	Young modulus	[36]
ν	1/3	Poisson ratio	‘’
ε_c_	200 µN/m	cell-cell anchorage	‘’
V_p_	0.88 V_0_	threshold volume of contact inhibition	Set
**Parameter of the BM model**			
z_0_	150 µm	length of the crypt	Set, according to measured properties of the crypt shape [8]
r_0_	60 µm	crypt radius at the crypt-villus junction	‘’
λ_1_	0.25	shape parameter 1	‘’
λ_2_	0.1	shape parameter 2	‘’
λ_MAX_	1.25 µm	maximum in-radius of a network triangle	Set (technical)
Ω	0.95	threshold ratio	Set
ε_K_^Paneth^	35 10^−12^ Nm	maximum cell-knot interaction energy of Paneth cells	ensuring apoptosis rates <5% [39]
ε_K_^other^	5.5 10^−12^ Nm	maximum cell-knot interaction energy of all other cells	
**Parameter of crypt dynamics**			
η_c_	5×10^10^ Ns/m^3^	friction constant for cell-cell friction	[36]
η_BM_	3.2 Ns/m	friction coefficient for cell-BM friction	Fit: turnover
η_VO_	400 Ns/m	friction coefficient regarding volume changes	[36]
F_A_^Paneth^	7.5 nN	absolute value of the migration force of Paneth cells	Fit: Distribution of Paneth cells
F_A_^other^	4.5 nN	absolute value of the migration force of all other cells	Fit: turnover and Brdu data
**Parameter of the lineage specification and differentiation model**			
z_p_	−125 µm	position of the Wnt- threshold TP_Wnt_ for priming	Fit: size of the Paneth cell compartment
z_d_	−87.5 µm	position of the Wnt- threshold TD_Wnt_ for differentiation	Fit: turnover and Brdu data
LP^Paneth^	0.35	Notch activation through Paneth cells	Fit: cell ratios
LP^Goblet^	1.00	Notch activation through Goblet cells	Set: maximum
TD_Notch_	1	Notch-threshold	Set
t_P_	57 days	lifetime of a Paneth cell	[1]

Steady state cell patterning in the model crypt results as a self-organized feature referring to the assumed modes of Notch- and Wnt-signalling. Undifferentiated functional stem cells, i.e. cells with high Wnt- and Notch-signalling are intermingled among the Paneth cells and appear up to cell position 4 and 5. Thus, they match the distribution of LGR-5 positive cells reported by Barker et al. [6] (compare Fig. 1a,c). They also comprise that of the Bmi1-positive subpopulation [7]. All undifferentiated cells are capable of proliferating. We found about 94% of them to divide within less than 48h. However, a fraction of about 1% of the undifferentiated cells remains quiescent for more than 1 week and thus carries features of label retaining cells [9].

The model generates a Paneth cell population at the crypt bottom whose spatial distribution fits the data observed by Chwanlinski et al. [40] (Fig. 2c). Interestingly, Paneth cells are predominately induced between position 2 and 5 (Fig. 2c, insert) and move down the crypt. This is in agreement with the experimental observations by Bjerknes et al. [41]. Moreover, a spatial distribution of Goblet cells is generated which is very similar to the one reported by Paulus et al. [42] (Fig. 2d).

#### Modelling clonal dynamics in the steady state

**Figure 3:**
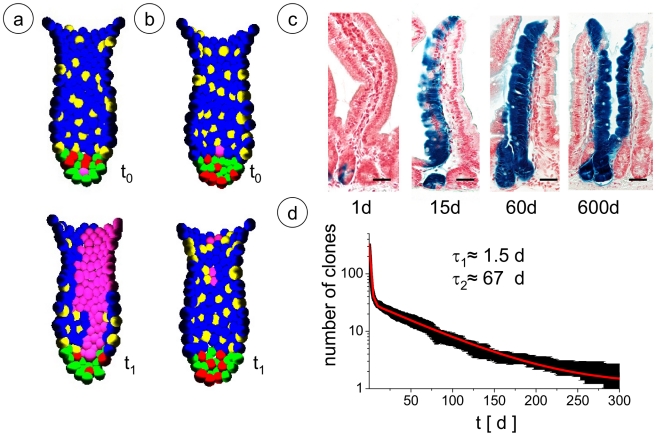
Clonal dynamics. a),b) Snapshots of simulated cell clones (pink) at labelling initiation (t_0_) and 7days later (t_1_) for clones derived from a) an undifferentiated functional stem cell and b) an enterocyte progenitor. Colour code as in Fig. 1. c) Clonal expansion in LGR5-EGFP-IRES-creERT2 knock-in mice crossed with Rosa26-lacZ reporter mice 1, 15, 60 and 600 days after tamoxifen injection [6]. The clones of LGR-5 expressing functional stem cells persist over long times. Bars: 50µm. d) Simulated clonal conversion in crypts: simulated data (black) and double exponential fit with time constants τ_1_ and τ_2_ (red). A video showing an example of a simulation of clonal conversion in a crypt can be found in Video S2.

Simulating steady state conditions, we studied the dynamics of clonal expansion and conversion to monoclonality. Technically, in the model all cells were labelled at one instance with a clonal marker that is inherited by all offspring. The number of coexisting clones was followed over time. In the long run, few clones survive and only one clone eventually populates the entire crypt. The life-span time of clones in the crypt depends on the initial position of the labelled cell. While clones of functional stem cells initially located at the bottom of the crypt are more likely to persist for a long time (Fig. 3a), the probability is dramatically reduced for progenitor cells at higher positions in the crypt (Fig. 3b) and drops to zero for terminal differentiated cells. Our observations on clonal coexistence and on conversion to monoclonality on a time scale of weeks (Fig. 3d, see also Video S2) are in agreement with experimental studies where random mutations of genetic clone markers were used [43], [44].

#### Modelling gain and loss of function mutants

**Figure 4:**
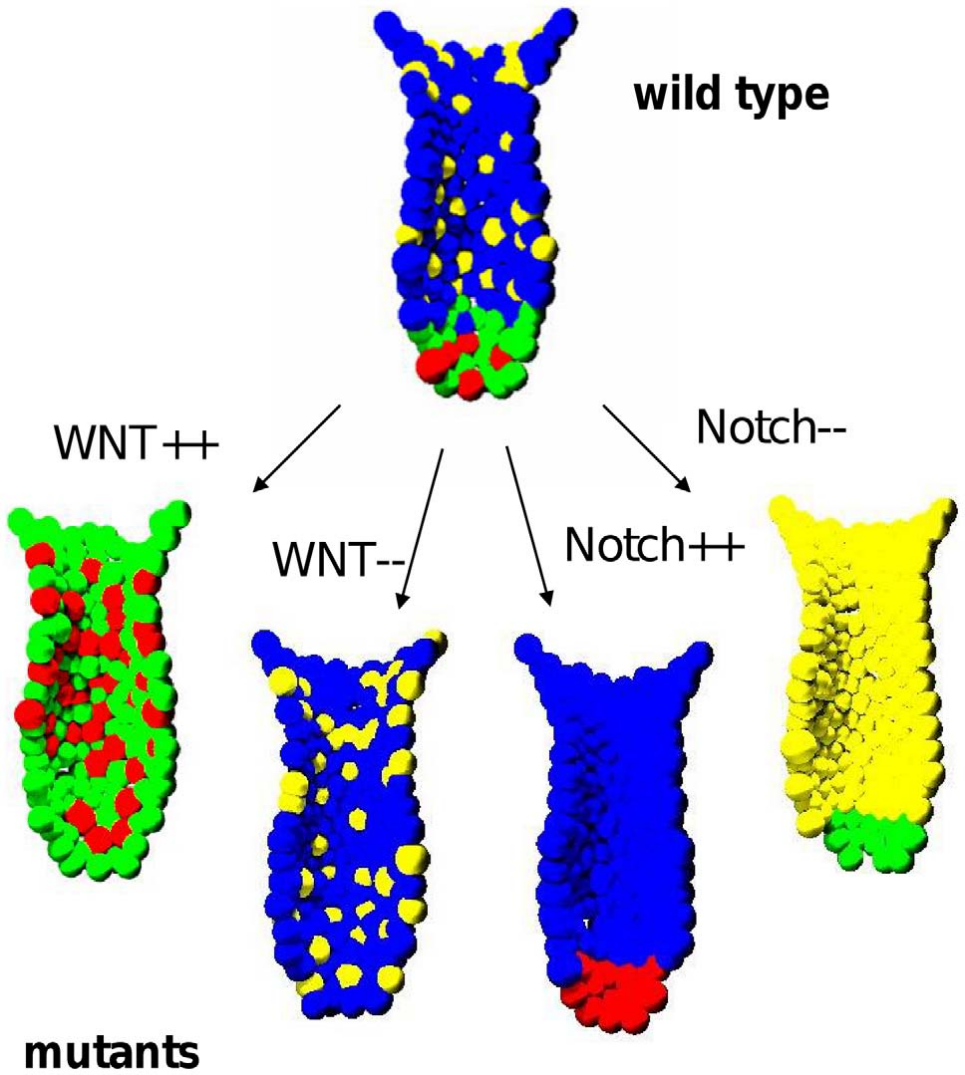
Gain and loss of gene function studies. Simulation results for crypt organisation following disturbed signalling. Colour code as in Fig. 1. **Wnt++:** Consitutive activation of Wnt in all cells leads to an expansion of the populations of undifferentiated and Paneth cells and a complete loss of Goblet and enterocyte progenitors. **Wnt--:** Reduced Wnt-signalling results in a complete loss of undifferentiated and Paneth cells. **Notch++:** Assuming constitutive active Notch-signalling in all cells completely suppresses the secretory lineages. **Notch--:** In contrast, a complete block of Notch-signalling results in full depletion of undifferentiated cells and the absorptive lineage.

Our model also permits a better understanding of the prompt effects of conditionally de-regulated Wnt- and Notch- signalling. A constitutive activation of the Wnt-signalling was implemented in the model by increasing the Wnt-activity over TP_Wnt_ in all cells along the crypt-villus axis. This results in a rapid expansion of the functional stem cell and Paneth cell population at the expense of the enterocyte and Goblet cell population (Fig. 4, Wnt++). In parallel, the turnover of the system decreases. This is in accordance with the experimental results obtained by Sansom et al. [34] and Andreu et al. [21] studying APC-loss in transgenic mice. In contrast, functional stem and Paneth cells vanish if the Wnt-signalling is kept below TP_Wnt_ in all cells (Fig. 4, Wnt−). However, proliferative cells still remain in the crypt. A comparable behaviour was obtained by Fevr et al. [33] studying tissue-specific, inducible β-catenin gene ablation in adult mice.

Constitutive activation of Notch-signalling in all cells above TP_Notch_ resulted in a rapid Paneth- and Goblet- cell depletion and an increased cell turnover (Fig. 4, Notch++). Such behaviour was observed experimentally by Fre et al. [31] targeting the expression of a constitutively active form of the mouse Notch 1-receptor in all cells of the intestinal epithelium. A complete inhibition of Notch-signalling in all cells in turn leads to a conversion of stem cells into Paneth progenitors and of enterocyte into Goblet progenitors (Fig. 4, Notch−). Over time this results in changed proliferation activity throughout the crypt. This behaviour is in agreement with the findings by van Es et al. [12] demonstrating that inhibition of Notch-signalling by use of a γ-secretase inhibitors or removal of the transcription factor CSL leads to rapid Goblet cell conversion throughout the crypts.

#### Robustness against elimination of subpopulations

An essential model feature is the reversibility and flexibility of cell fate decisions in cells not yet terminally differentiated. In principle we give all enterocyte progenitors the capability of acting as functional stem cells if they enter the spatial Wnt-niche at the crypt bottom. Likewise, cells primed to become Paneth or Goblet cells may revert their status depending on the local Notch and Wnt-signals. Although possible in principle, such cell fate reversions are only occasional events in steady state (e.g. less than 0.01 transitions per enterocyte progenitor into the undifferentiated state per day). They can, however, become more relevant in states of perturbation.

This concept implies that the loss of a single cell or of a few cells is immediately compensated for by neighbouring cells which can rapidly adapt due to local signalling and thereby provide robustness against tissue perturbations. We analysed the model robustness to a sudden elimination of selected cell populations from steady state conditions. In independent simulations we separately deleted i) all undifferentiated cells, ii) all cells committed to the Paneth- cell lineage and iii) to the Goblet-cell lineage and finally iv) all enterocyte progenitors. A complete deletion of the undifferentiated cells at the crypt bottom only transiently affects the crypt system. This is in agreement with observations following the loss of functional Lgr5 stem cells upon conditional deletion of the stem cell-specific transcription factor ASCL2 [45]. In our simulations the steady state recovered after about 5 days following de-differentiation events of progenitor cells. At about the same time the Paneth cell pool was completely replenished following its deletion. Thereby, the increased commitment of undifferentiated cells into the Paneth cell lineage did not affect the number of undifferentiated cells. Regeneration of the Goblet cell and enterocyte compartment was also achieved within approximately 2 days and 4 days, respectively. Here, deletion of one compartment leads to a temporal increase of the other. In summary, our model predicts that each type of functional cells within the crypt could be transiently deleted, without the appearance of long-term adverse effects on crypt homeostasis. Only a deletion of the entire proliferative compartment, i.e. all undifferentiated and progenitor cells, eventually stops regenerative action and ‘kills’ the model crypt. Assuming irreversible fate decisions this kind of robust regeneration vanishes.

## Discussion

The spatio-temporal organisation of the intestinal epithelium has been modelled using several approaches [46], [47], [48], [49]. The model presented here represents the most comprehensive model of intestinal stem cell and tissue organization proposed so far. It links a broad range of observed phenomena into one conceptual framework.

The model is based on single cells acting as individual agents, updating their status within a certain set of options governed by some active rules and on signals received from the environment. Thereby, it accounts for and requires the 3-D spatial structure of the crypt. The model describes how cell production and cell fate decisions could be organized in steady state as well as under perturbations. Thus, the model offers a novel systems biological view on crypt stem cell and tissue organisation.

Recently, Lgr-5 and Bmi-1 have been identified as markers linked to functional stem cells in the small intestine [6], [50]. In our model no stem cell population was implied. However, the Lgr-5 positive subpopulation can be projected to the undifferentiated cell population at the very bottom of the crypt in our model system, although it may be not identical. Bmi-1 positive cells could also be associated to the subpopulation of functional model stem cells which approach the Wnt-activity threshold TP_Wnt_. We found that the behaviour of all these cells is in full accordance with the functional definition of stem cells [8].

Our simulation results suggest that any single subpopulation of the crypt could be deleted at a certain time point without any long term consequence for crypt organisation. While this prediction remains to be validated experimentally, it does raise additional questions regarding the origin of this kind of robustness. In our model, robust organisation of the intestine depends on the assumption of i) reversible and flexible fate decisions of stem cells and ii) an ‘externally’ defined Wnt-activity gradient.

There is increasing evidence of reversible and flexible fate decisions from other tissue modelling studies [51], [52], [53]. However, direct evidence for this phenomenon in the intestinal tissue is still missing. It could be provided for de-differentiation events by an experiment involving cell tracers. Tracing e.g. a cell which is already committed to the Paneth property, one would not observe the expansion of a large clone in case this fate decision is irreversible. In case the cell is capable of switching back into an undifferentiated state there is a certain probability to observe this event. This probability could be increased applying strong perturbation as e.g. radiation. Fate switches of a cell between different progenitor states would require continuous single cell imaging [54]. The in vitro organoid system introduced by Sato et al. [22] in principle enables such investigations.

Our assumption of the dependence of the Wnt-activity on local curvature of the tissue rises the question on the underlying molecular regulation. A possible link between surface curvature, β-catenin stabilisation and enforced Wnt-signalling could be provided for example by integrin-linked kinase activity [55], [56]. Moreover, experimental results demonstrated that Wnt-signalling is required for apical constriction of epithelial cells during gastrulation in C. elegans [57]. It was hypothesised that this function is widely conserved in higher species [58]. Our line of argument would implicate a bi-directional relationship between local curvature and Wnt-expression, with Wnt-activity inducing positive curvature and positive curvature enforcing Wnt-activity. Such dependence between morphogenes and tissue curvature was in fact suggested by Cummings [59] in a theoretical framework. As a result the intestinal crypt shape would be a self-organised feature. Actually, loss of Paneth cells in SOX9 deficient mice results in a changed crypt diameter [20] suggesting Paneth cells to induce local curvature.

A recent study by van Leeuwen et al. [48] focused on Wnt-signalling in the intestinal crypt. By combining models of molecular regulatory networks of Wnt-signalling and cell-cycle progression with a biomechanical model of the crypt epithelium they demonstrated that an extended Wnt-gradient along the crypt-villus axis is not required in order to explain the proliferative pattern in the crypt. They authors linked proliferative activity in the upper crypt to delays in Wnt-signalling. This opens the question whether terminal post-mitotic differentiation of enteroccytes and Goblet cells is actually related to an external signal that the cells receive at a particular position or is the consequence of a maturation process that starts already with priming. We here assumed an external signal. Actually, this fate decision may not only involve the Wnt-pathway [25].

Our model predicts that many more cells than the actual functional stem cells at the crypt base can be clonogenic but that the probability a certain clone overtakes the entire crypt depends on the position of its initiation. In the model, this probability is directly related to the probability that the progeny of the clone reaches the crypt bottom. This prediction also remains valid for systems with de-regulated signalling. Clonal expansion of individual APC-mutant cells was recently shown to be effective for Lgr-5 positive cells restricted to the crypt base [60]. This is in full accordance with our predictions.

Long living clones were suggested to profit from specific environmental interactions such as interaction with enteroendocrine cells expressing growth inhibitory peptides [61] or with specialised stroma cells, so-called myofibroblasts expressing e.g. BMP antagonists [62]. The impact of all these interactions on the system dynamics are not considered in our present model. Also effects like the potential co-regulation between active and quiescent subpopulations of functional stem cells [63] are beyond the scope of our model. We focussed on Wnt- and Notch-signalling as independent key regulators. Cross-talk [27], [64] has not been considered. Interestingly, inhibition of BMP-signalling at the villus can lead to crypt *de novo* formation by crypt fission and adenoma polyp growth [65]. Our model is designed to encompass such spatial dynamics. However, this will require incorporating a model of basal membrane reorganisation, which is currently in preparation. This model extension will permit to simulate scenarios of crypt formation, crypt budding and adenoma formation as a consequence of self-organised crypt shape.

The model proposed in this study comprehensively explains numerous experimental observations regarding spatial patterns of proliferation, clonal dynamics, cell lineage specification and differentiation under both normal steady state and disturbed regulation. Thereby, it combines features of the molecular, cellular and tissue levels, providing a simplified but consistent picture of the dynamic organisation of small intestinal crypts. We expect that the model can be specified according to the specific organisation of duodenum, jejunum, ileum and colon crypts by adapting specific parameter sets. The predictions provided in this study can be validated experimentally. Thus, we expect that our novel approach will provoke further discussion about somatic stem cell organisation and will stimulate future experimental and modelling research in the field.

## Materials and Methods

In the following we provide some details regarding the model, the fitting of experimental data, and experimental setups. In section A1) and A2) we describe the cell-cell and the cell-basal membrane (BM) interaction model, respectively. In section A3) the equations of motion are given and the update procedure of the internal state vector of the cells is explained. In section A4) the fitting strategy is described and some results on the effects of parameter variations are discussed. Finally, in section A5) material and methods of the BrdU labelling experiments and details of its simulation are provided.

### A1: Cell model and cell-cell interactions

An isolated cell is represented by an elastic sphere of radius R and volume V(R). If a cell i gets into contact with another cell j the cells adhere. Their adhesive interaction energy is approximated by:
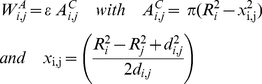
(A1)where ε denotes the adhesion energy per unit contact area and 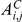 the contact area of the cells. 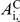 depends on the radii of the cells R_i_, R_j_, and on the distance d_i,j_ between them. As a result of contact formation the shapes of the cells change by flattening at the contact area. Assuming that cells can be described by an isotropic homogenous elastic solid, the deformation energy for the contact is calculated using the Hertz model:
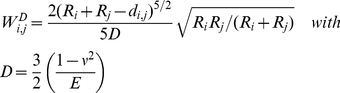
(A2)Here, E is the Young modulus and ν the Poisson ratio of the cells. Additional to the shapes the volumes of the cells change during contact formation as well. The energy related with a volume change of a cell is approximated by the energy of a uniform compression (or inflation) of the spheres assuming a bulk modulus K:
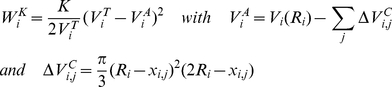
(A3)Here, 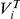 is the target volume, i.e. the volume the cell would adopt if it were isolated. 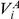 is the actual volume of the cell which depends on its radius and the individual volume overlaps 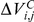 with all neighbouring cells (j).

### A2: BM model and cell-BM interactions

In our approach the BM is modelled by a triangulated fiber network. This network is represented by its knots. These knots are assumed to be located at the crypt surface, which is defined by the following equation for the local crypt radius:
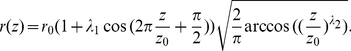
(A4)Here z_0_ is the length of the crypt and r_0_ the radius of the crypt at the crypt-villus junction (z = 0). The parameters λ_1_ and λ_2_ are shape parameters. A further parameter of the network is the maximum inradius (mesh size) of its triangles λ_MAX_. It was set narrow enough to avoid that cells can cross the network. In all simulations presented we used λ_MAX_ = 1.25 µm, corresponding to about 30.000 knots within the network of one crypt. On one hand this setting ensures low local variance of the network structure in terms of the coordination number. On the other hand it keeps the system computational tractable.

If the distance between a cell i and a knot k of the BM network d_i,k_ is smaller then the radius of the cell R_i_ they are assumed to interact. The interaction energy is modeled by:
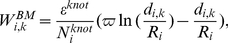
(A5)where *ε^knot^* denotes the maximum interaction energy per knot. While for d_ij_ larger then 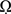
R_i_ the interaction is weakly adhesive, it becomes strongly repulsive for d_ij_ smaller then this threshold distance. The interaction energy is scaled by the number of knots 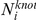 interacting with cell (i). This number has an upper limit depending on the ratio between cell and mesh size. Decreasing 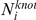 strengthens the interaction with the individual knots and thus, the total interaction of the cell with the network remains largely unaffected.

### A3: Cell motion and changes of the internal state vector

The generalized forces acting on cell i can be derived from the partial derivative of the interaction energies described above:
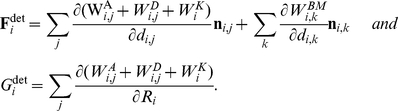
(A6)The distance d_ij_ is given by d_ij_ = |**r**_ij_| = |**r**_i_−**r**_j_| where **r**_i_ and **r**_j_ are the position vectors of cell i and j, respectively. In the same way r_i,k_ is the distance between cell i and the knot k. **n**_i,j_ = **r**_ij_/|r_ij_| and **n**_i,k_ = **r**_i,k_/|r_i,k_|.

These forces organize the contacts between the cells by changing their distance or their radii. The resulting cell motion can be modeled using Langevin equations for each cell [36]. However, throughout this study we neglected fluctuations. Moreover, the small Reynolds numbers in the regime of single cells allowed us to neglect their inertia. Thus, cell motion was described by a system of linear differential equations, where the displacement d**r**_i_ and the radius change dR_i_ of cell i are given by:
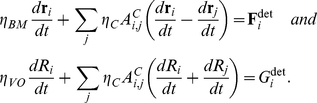
(A7)The sums run over all neighboring cells j in direct contact to cell i. The friction coefficients η_BM_ and η_VO_ describe friction between a cell and the BM and in course of volume changes, respectively. The friction between two cells is described by the coefficient 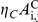 which is proportional to the contact area.

During each time step of a simulation position and radius of all cells are updated in parallel according to equation (A7). Thereby, a variable time step is used in order to avoid artificial cell interpenetration. Each update of position and radius potentially changes the internal state vector 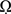 (Wnt- and Notch- activity, I_Wnt_, I_Notch_) of the individual cells. Thus, 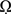 is updated subsequently. Thereby, the activity I_Wnt_, I_Notch_ may cross one or more threshold values (TP_Wnt_, TD_Wnt_, TD_Notch_). In this case the phenotype of the cells changes and all properties of the new phenotype - including the lineage characteristics, as well as migration and adhesion properties - are assigned to the cell.

In our model Wnt-activity is assumed to be a function *f* of the local curvature of the BM which itself is a function of the position along the crypt axis z (see Fig. 1):
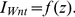
(A8)We set the threshold values of the Wnt-activity TP_Wnt_ and TD_Wnt_ equal to *f*(z_p_) and *f*(z_d_) for lineage priming and terminal differentiation, respectively. Accordingly, changes of the cell fate occur if a cell crosses the position z_p_ or z_d_. We used these threshold values as fit parameters to adjust the systems behavior (see A4 and Table 1). After fitting the model, the positions z<z_p_ cover the regions of high curvature. Moreover, at positions z_p_<z<z_d_ both types of curvature are positive and nearly constant and at positions z>z_d_ the Gaussian curvature falls below zero. Thus, the fitting results are in full accordance with our assumptions of a correlation between Wnt-activity and positive curvature.

The Notch-activity is calculated via cell-cell contact analysis. A cell is Notch-activated by all cells being in direct contact with it and expressing Notch-ligands:
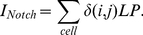
(A9)Here, the sum runs over all cells of the crypt. δ(i,j) is equal to one if R_i_+R_j_>d_ij_ (condition of direct contact) otherwise it is zero. The degree of activation by a single cell (LP) depends on the cell type. LP is assumed to be larger than zero for Paneth and Goblet cells and zero for all other cells. In order to reproduce the correct cell patterning Paneth cells are required to induce weaker activation than Goblet cells (see A4 and Table 1). A cell changes its fate if its Notch-activity crosses the threshold TD_Notch_ (see also Table S3).

### A4: Parameter variations and fitting strategy

In our model we assume that the Wnt-activity of the individual cells is determined by the local curvature of the basal membrane. Thus, the crypt geometry impacts the lineage specification and differentiation and consequently the crypt turnover. In order to study these interrelations we set the shape parameter λ_1_ to zero and varied the crypt length and width. By assigning the thresholds TP_WNT_ and TD_WNT_ fixed Gaussian curvatures 4×10^−4^/µm^2^ and 0/µm^2^, respectively, the shape changes resulted in a shifted position of these thresholds along the crypt axis. We found that the shape changes did result in quantitative changes of the systems behavior only. Selected results can be found in the Table S1. In all further simulations we considered a defined cell shape that agrees with experimental data on crypt geometry [8]. The crypt with this defined shape is called ‘reference crypt’ in the following. The parameters of the reference crypt are listed in Table 1.

In a series of simulations we varied the threshold TP_Wnt_ for a reference crypt (see Table S2). In this case the total number of cells remains approximately fixed. The changes result in changes of the size of the Paneth cell compartment (undifferentiated and Paneth cells) which are balanced by changes of the number of enteroytes and Goblet cells. A decrease of the size of the Paneth cell compartment increases the number of proliferative cells in the crypt and thus decreases the turnover time. We selected the position of the threshold TP_Wnt_ such that cell number of the Paneth cell compartment is about 40 [40].

For a given position of TP_Wnt_ the steady state cell production of a crypt still depends on cell interaction parameters as well as internal parameters regulating fate decisions. For example the turnover is decreased as a result of an increase of the cell-cell interaction strength ε_c_, an increase of the sensitivity to contact inhibition V_p_ or a decrease of the Wnt-activity threshold TD_Wnt_. We used TD_Wnt_, together with F_A_^other^ and η_BM_, to fit the turnover the results of the BrdU labelling experiments.

This was most efficient provided that the average apoptosis rate in the crypt was smaller than about 5% per day. Such low apoptosis rates were ensured assuming a high cell-knot interaction constant ε_k_>5 nNm for all cells. Note that a migration force F_A_^other^>0 was required to fit the BrdU labelling data.

**Figure 5:**
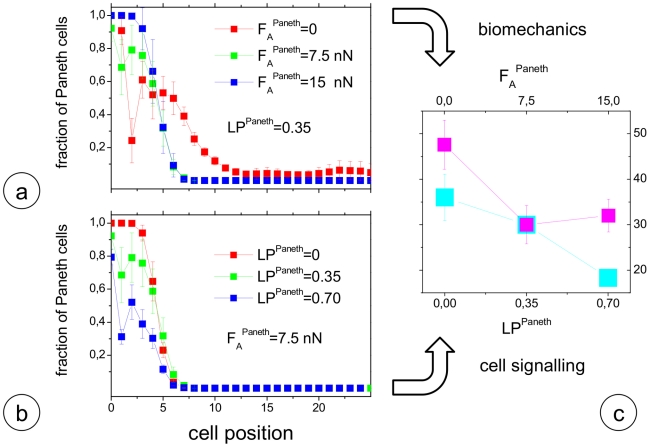
Paneth cell distribution and numbers are affected by biomechanics as well as cell signalling. a) Local fraction of Paneth cells in dependence of their migration force F_A_^Paneth^. b) Local fraction of Paneth cells in dependence of their Notch-activation strength LP^Paneth^. All other parameters of the model are fix (see Table 1). c) Average total number of Paneth cells as obtained in simulations changing F_A_^Paneth^ (pink squares, compare a)) and changing LP^Paneth^ (cyan squares, compare b)).

Steady state cell patterning also underlies a complex regulation as seen from the organisation of the Paneth cell population. For a given crypt geometry and Wnt-activity threshold TP_Wnt_ the sum of undifferentiated and Paneth cells is approximately fixed. Thereby, the number of Paneth cells depends sensitively on the cell-knot interaction strength ε_k_^paneth^, the migration force for Paneth cells F_A_^Paneth^ and the Notch-activation strength LP^Paneth^. Stable Paneth cell adhesion to the BM over their life time t_p_ required ε_k_^paneth^≥35 nN defining a constraint to this parameter. Moreover, a minimum ‘migration force’ F_A_^Paneth^ of about 7nN is required to ensure that Paneth cells remain confined at the crypt bottom. Thus, we adjusted the number of Paneth cells using LP^Paneth^ (Fig. 5).

### A5: BrdU labeling experiments and simulation

#### Experiments

To examine proliferating cells 2 mg/ml BrdU (Sigma-Aldrich, Deisenhofen, Germany)/PBS was injected i.p. into mice (50mg/kg b.w.). Mice were sacrificed 2 and 24 hrs after injection. The bowel segments were fixed in 4% paraformaldehyd/PBS and paraffin embedded. In 4µm sections proliferating cells were detected after blocking endogenous peroxidase activity in 3% H_2_O_2_/PBS for 10 min, 2N HCl DNA denaturation for 30 min and enzymatic pretreatment with 0.1% (w/v) Trypsin (SIGMA) for 20 min by incubating with an anti-BrdU monoclonal antibody (Sigma) for 2 hrs (all steps at 37°C) followed by the Vectastain® ABC kit (Vector Laboratories, Burlingame, US). Finally, sections were counter-stained with hematoxylin, dehydrated and mounted. Fifty half-crypts per mouse were scored on a cell positional basis according to whether or not cells were BrdU positive.

#### Modeling

These experiments were simulated assuming that 70% of the proliferating cells were marked. This number was chosen somewhat larger than the fraction of the cell cycle time belonging to the S-phase (50–60%^37^), accounting for an extended labeling time. Labels were inherited to the entire progeny. Sections of 4.5µm were analyzed.

## Supporting Information

Table S1 Simulation results on the impact of the crypt shape on the systems behaviour. For a simple crypt shape (λ_1_ = 0) the length and width of the crypt was changed, by changing the parameter z_0_ and r_0_. The thresholds TP_Wnt_ and TD_Wnt_ were set to the positions of Gaussian curvature 4×10^−4^/µm^2^ and 0/µm^2^, respectively (see black lines). Increasing length increases the number of cells leaving the crypt thereby the turnover time remains approximately constant. Increasing width increases the turnover time, i.e. the outgrowth is less efficient.
(0.55 MB TIF)

Table S2 Simulation results on the impact of the position of the threshold TP_Wnt_ on the systems behavior. Moving down the position of TP_Wnt_ (black lines) to the crypt bottom leads to a faster turnover. This refers to a decreasing number of Paneth cells which is mainly balanced by proliferative enterocyte progenitors. For positions x_p_>x_0_ the system resembles the situation of a Wnt− system discussed in the text (Fig. 3).
(0.47 MB TIF)

Table S3 Simulation results on the impact of the threshold TP_Notch_ on the systems behaviour. Increasing the threshold leads to an increased number of secretory cells in the crypt at the expense of undifferentiated cells and enterocyte progenitors. Note that the number of Goblet cell increases only if TP_Notch_ becomes larger than 1 due to discrete numbers of neighbour cells. At a certain value of TP_Notch_ stimulation by the neighbour cell is no longer sufficient and all cells will turn on secretory fates. In this case the system resembles the situation of a Notch− system discussed in the text (Fig. 3).
(0.48 MB TIF)

Video S1 Example of a simulation of a steady state crypt. Undifferentiated cells (red) and Paneth cells (green) are found intermingled at the crypt bottom, progenitors of enterocytes (blue) and Goblet cells (yellow) move upwards the crypt axis. One second of the video represents 1.25 days.
(3.03 MB AVI)

Video S2 Example of simulated clonal conversion in a crypt. A labelled cell clone (pink), expands in the crypt. It originates from an undifferentiated functional stem cell. Colour code of crypt cells as in Video S1. One second of the video represents 1.25 days.
(2.58 MB AVI)
