# Supplementary figures and images for: A Comprehensive Model of the Spatio-Temporal Stem Cell and Tissue Organisation in the Intestinal Crypt
Source: PLoS Comput Biol. 2011 Jan 6;7(1):e1001045. doi: 10.1371/journal.pcbi.1001045 (PMC3017108; doi:10.1371/journal.pcbi.1001045)

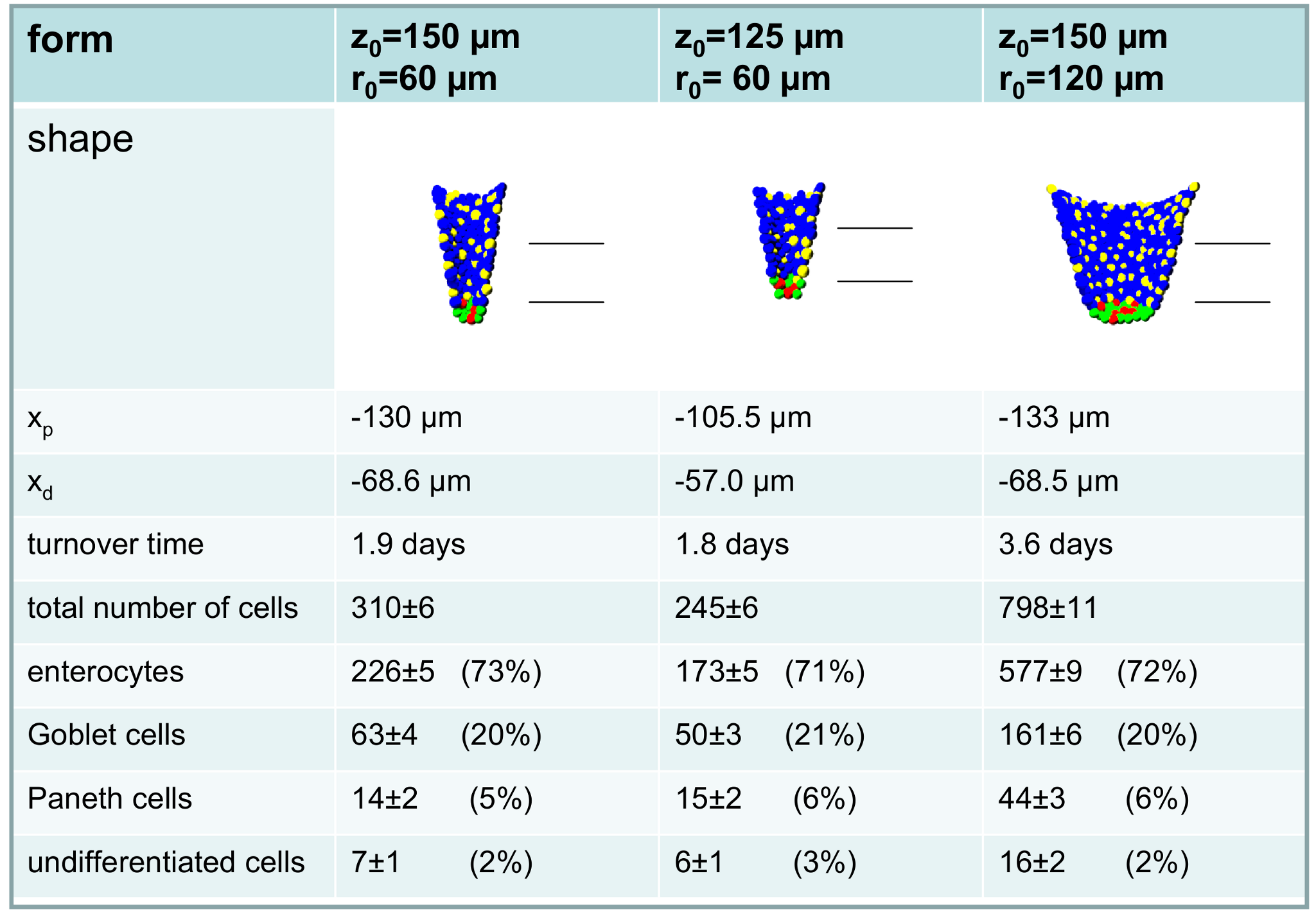

Supplement: Table S1 — Simulation results on the impact of the crypt shape on the systems behaviour. For a simple crypt shape (λ1 = 0) the length and width of the crypt was changed, by changing the parameter z0 and r0. The thresholds TPWnt and TDWnt were set to the positions of Gaussian curvature 4×10^−4^/µm2 and 0/µm2, respectively (see black lines). Increasing length increases the number of cells leaving the crypt thereby the turnover time remains approximately constant. Increasing width increases the turnover time, i.e. the outgrowth is less efficient. (0.55 MB TIF) [file pcbi.1001045.s001.tif]

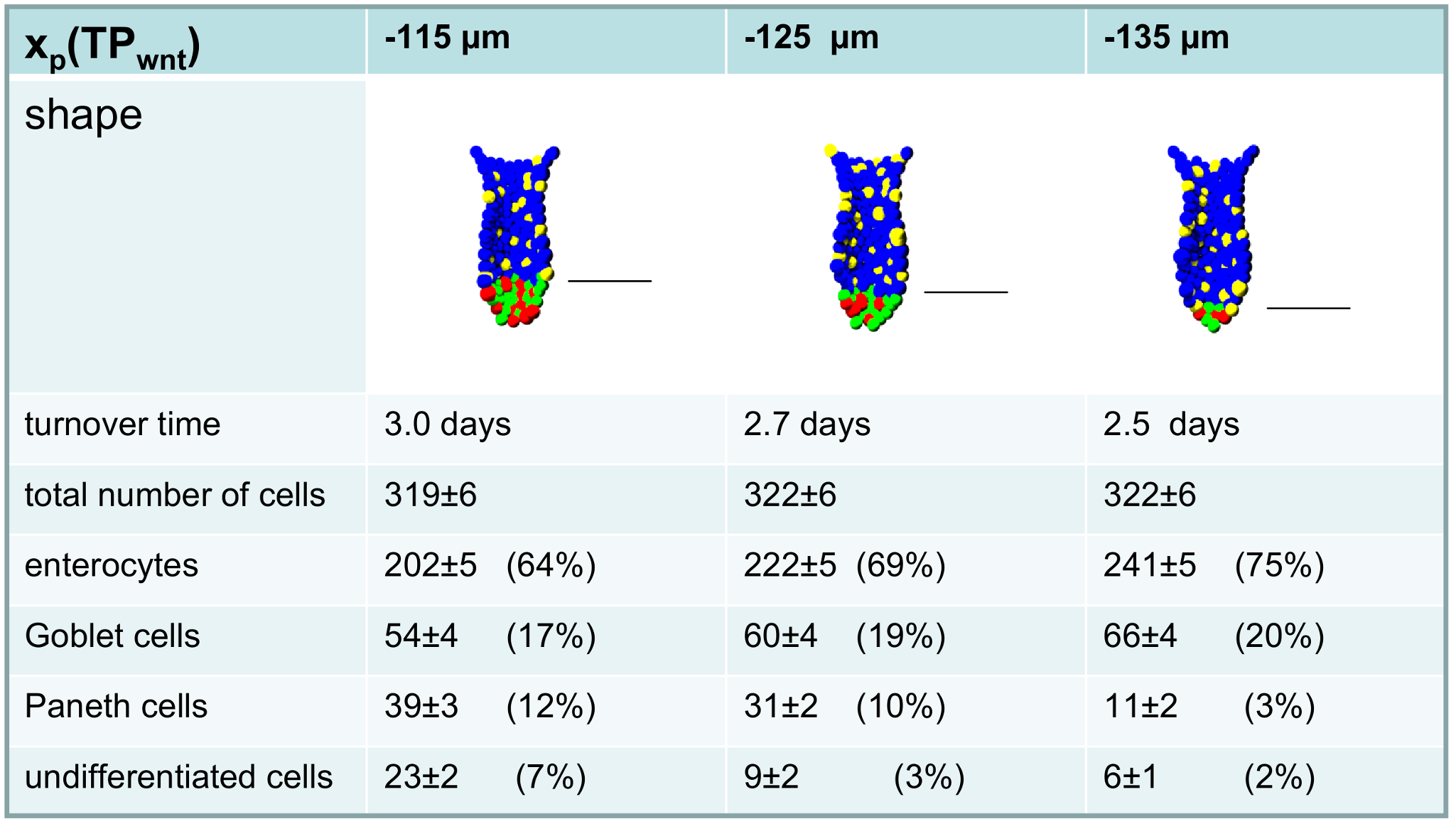

Supplement: Table S2 — Simulation results on the impact of the position of the threshold TPWnt on the systems behavior. Moving down the position of TPWnt (black lines) to the crypt bottom leads to a faster turnover. This refers to a decreasing number of Paneth cells which is mainly balanced by proliferative enterocyte progenitors. For positions xp>x0 the system resembles the situation of a Wnt− system discussed in the text (Fig. 3). (0.47 MB TIF) [file pcbi.1001045.s002.tif]

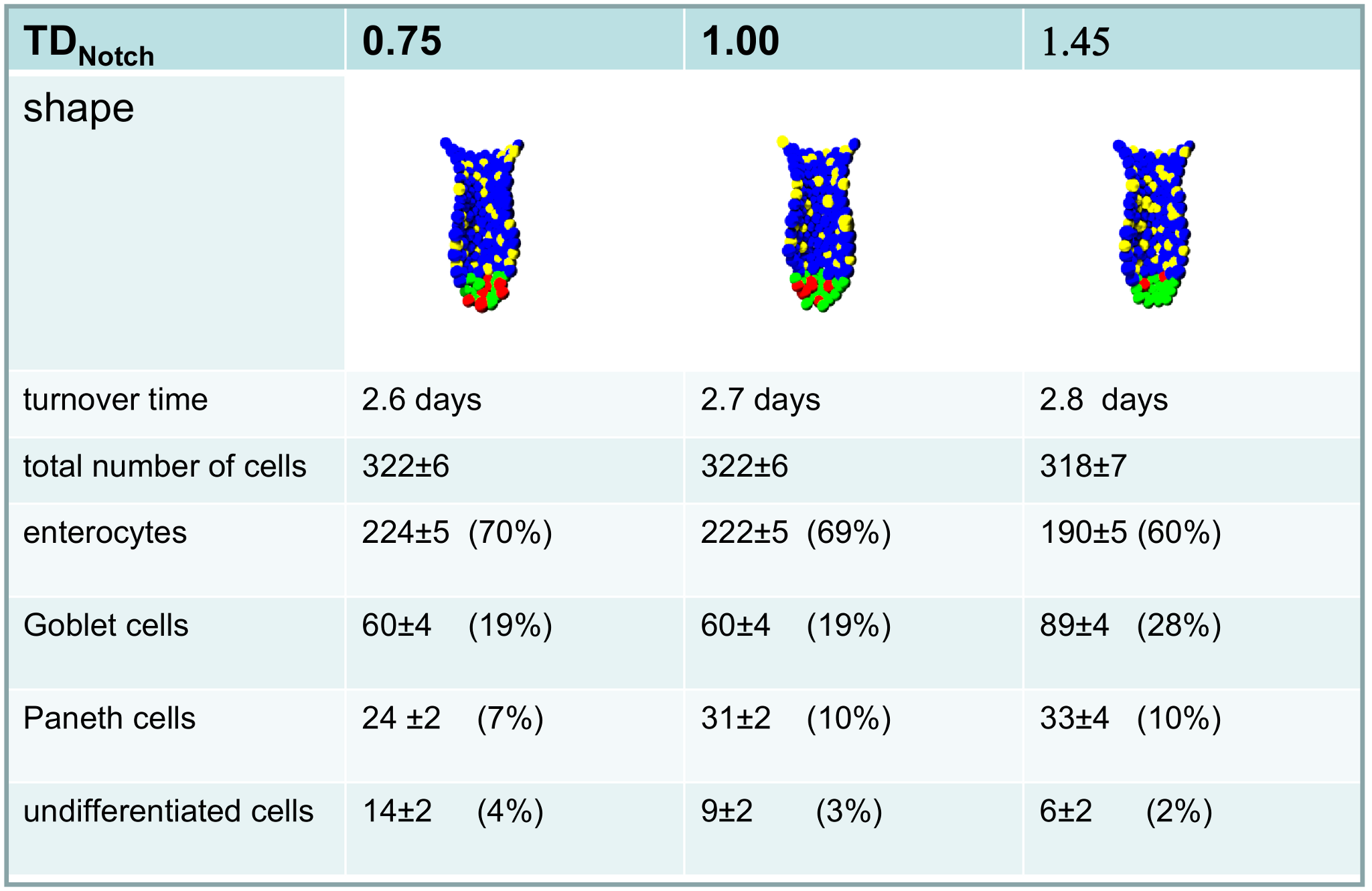

Supplement: Table S3 — Simulation results on the impact of the threshold TPNotch on the systems behaviour. Increasing the threshold leads to an increased number of secretory cells in the crypt at the expense of undifferentiated cells and enterocyte progenitors. Note that the number of Goblet cell increases only if TPNotch becomes larger than 1 due to discrete numbers of neighbour cells. At a certain value of TPNotch stimulation by the neighbour cell is no longer sufficient and all cells will turn on secretory fates. In this case the system resembles the situation of a Notch− system discussed in the text (Fig. 3). (0.48 MB TIF) [file pcbi.1001045.s003.tif]
